# Children's awareness of ethnic outgroup symbols: Piloting a task in the Republic of Ireland

**DOI:** 10.1002/ijop.12870

**Published:** 2022-06-26

**Authors:** Dearbháile Counihan, Sarah Carol, Laura K. Taylor

**Affiliations:** ^1^ School of Psychology, University College Dublin Dublin Ireland; ^2^ School of Sociology University College Dublin Dublin Ireland; ^3^ School of Psychology University College Dublin/Queen's University Belfast Dublin Ireland

**Keywords:** Children, Ethnic awareness, Marginalised group, Travellers, Ireland

## Abstract

Exploring children's awareness of social categories could uncover the foundation of intergroup attitudes and behaviours. Indigenous to Ireland, Travellers are an ethnic minority marked by a tradition of nomadism, only formally recognised as a distinct ethnic group in 2017. This brief report analyses data from 148 children aged 6–12 (55% female) in the Republic of Ireland. A quantitative task was adapted and applied to assess children's awareness of symbols associated with the Traveller community. We found that primary school children could accurately categorise the symbols which relied on perceptually obvious markers of Traveller identity, and that this remained stable across middle childhood. However, children did not correctly categorise symbols related to less observable elements (e.g., language, trade, religion). To the best of our knowledge, these findings are the first to identify specific symbols which are salient in children's awareness about this marginalised group. Implications for school‐based interventions are discussed.

From infancy, children form various categories (e.g., moms, babies) as they navigate their social worlds (Rhodes, 2013). Social categorisation supports children in organising details about people in their environment as they are classified by common demographic features, social roles, kinship networks, shared duties and other social cues (Bodenhausen et al., 2012). Through an internalisation of these categories, awareness of relevant groups exerts a substantial influence on children's attitudinal and behavioural responses. Ethnic awareness is the first stage of Social Identity Development Theory (SIDT; Nesdale, 2004). Quintana (1998) observed differing levels of ethnic awareness with age; children aged 3–6 years relied solely on observable features (e.g., hair, skin colour). From 6–10 years, there was a shift in comprehension as school‐aged children beganto recognise non‐observable elements of ethnicity (e.g., language, customs, food preferences). Considerably less is known about when children classify social groups with no phenotypical differences (Tomovska Misoska et al., 2020). In such contexts, symbols associated with the ingroup/outgroup become particularly salient (Taylor et al., 2020; Tomovska Misoska et al., 2020). The present study fills this gap by investigating children's awareness of Travellers.

## THE TRAVELLER COMMUNITY

Travellers are an indigenous minority group that have inhabited Ireland for centuries (Keogh et al., 2020). Approximately 31,000 Travellers reside in the Republic of Ireland, comprising 0.7% of the population (Central Statistics Office, 2016). Although Travellers are native to Ireland, they are recognised as a distinctive ethnic group from the “settled” majority population (Keogh et al., 2020). Travellers share features of the majority culture, such as physical appearance, White skin colour, use of English and practice of Catholicism; more subtle variations within their culture and lifestyle differentiate them from the settled Irish (McElwee et al., 2003). Travellers are traditionally nomadic, constituting an integral aspect of their culture. Traveller identity is further characterised by a common history and experience, extended family networks, practices of self‐employment, tinsmithery and horse‐trading, and use of traditional language “Cant” (King & O'Riordan, 2019; McElwee et al., 2003).

Travellers experience severe levels of prejudice; they are among the most discriminated against groups in Europe, with 65% reporting ongoing discrimination (EUAFR, 2020). Travellers are marginalised within Irish society, facing extreme social exclusion and disadvantage across education, healthcare, employment and housing (Boyle et al., 2020; Keogh et al., 2020). From a young age, Travellers encounter significant prejudice within primary‐level education. Children are at risk of poor attendance and low educational attainment due to institutional discrimination (e.g., inadequate consideration of Traveller culture on the curriculum), alongside direct discrimination and bullying (Bloomer et al., 2013; Boyle et al., 2020).

Limited research has investigated settled children's awareness of Travellers. In a qualitative study, Devine et al. (2008) concluded that children understood minority ethnic groups through “non‐Irish” categorisations related to differences in skin colour, lifestyle, language and religious practices. Most participants only referenced Black children in their discussions of racism, suggesting that skin colour is salient in their self‐categorisation as White Irish. Children did not identify Travellers as a distinct ethnic group. Although name‐calling of Black peers was deemed racist by most children, name‐calling of Travellers based on cultural differences (e.g., modes of speech/dress, nomadic lifestyle) was not considered wrong.

Dupont (2017) observed that 8–10 year olds (*n* = 188) had limited personal contact and/or familiarity with Travellers. While over 70% of children in the study had previously heard of Travellers, only 15% had previously met a Traveller. In interviews, 35% of children referenced housing/living arrangements (e.g., caravans, halting sites, nomadic lifestyle) and 11% mentioned Traveller weddings; only a handful of children referenced less observable features of Traveller identity such as language (2%) and religion (<1%). Dupont (2017) postulates that, in the absence of perceptible symbols, it is unlikely children would be capable of distinguishing Travellers from settled people.

## CURRENT STUDY

The need to expand existing social categorisation research to include Travellers is evidenced by the prevalence and implications of negative attitudes towards this group and their exclusion, along with findings that majority‐group children are seemingly unaware of Traveller's ethnic minority status. Travellers are pertinent to study as, although they are similar in appearance, they are distinguishable from the settled Irish on account of less observable cultural differences (McElwee et al., 2003). Therefore, it is important to examine whether existing theories accurately encapsulate how majority‐group children learn about and behave towards Travellers. The aim of the current study is to adapt and apply a quantitative task that can be used to explore ethnic awareness of a variety of symbols associated with the Traveller community. Given scant previous research, there is no explicit hypothesis about which symbols will hold greater salience. Rather, overall ethnic awareness across a range of symbols will be explored to determine the categories (i.e., culture, housing, politics, language) that majority‐group children use to classify the Traveller community. The influence of age on children's ethnic awareness of Travellers will also be examined.

## METHOD

### Participants

The total sample comprised of 148 children aged 6–12 (*M* = 8.80, *SD* = 1.84; 55% female). For the quantitative task, power calculations in G*Power indicated that for a two‐tailed, one‐sample *t*‐test, *n* = 37 could detect between a medium and large effect size of .61, at α = .05 and power = .95 (Cohen, 1992). Therefore, we presented children with a random subset of five of the 20 possible image pairs (i.e., each image pair was viewed by approximately 25% of the total sample, *n* = 37). Most children identified as Irish (98.6%) and/or were born in Ireland (75%). Children were recruited from seven Facebook “parenting groups” in the Greater Dublin Area (covering Dublin and the counties of Meath, Kildare and Wicklow) from January to June 2021. Parents consented by following a link to an online information letter and consent form via Qualtrics. Once informed consent had been obtained, parents could schedule a suitable Zoom meeting time. Verbal assent was obtained from each child prior to data collection. As compensation for their participation, each child received a certificate of participation and a €5 online book voucher. Ethical approval was granted by University College Dublin [Number: 2020–24] with the 1964 Helsinki Declaration and its later amendments or comparable ethical standards.

### Procedure

Participants were assessed individually by a trained experimenter for approximately 20 minutes. Six undergraduate and graduate students were trained as part of the *Helping Kids*! lab and followed a standardised script. Preliminary analyses did not detect any significant differences in participants' responses across experimenters.^1^ The “screensharing” feature on Zoom was used to present the Qualtrics survey to each child; experimenters recorded their oral responses.

### Measures

#### Task development

A quantitative task was adapted and applied to explore the majority group children's awareness of symbols associated with the Traveller and settled communities. The structure of this task was adapted from previous studies exploring children's awareness of different social groups (Taylor et al., 2020, 2021; Tomovska Misoska et al., 2020), while the content (Traveller/settled images) was modified to the Irish context. The task comprised 20 pairs of hypothesised ingroup, and outgroup symbols derived from previous qualitative studies (Devine et al., 2008; Dupont, 2017; see Appendix C). For example, since nomadism was a salient cue that children associated with the Traveller community, one image depicted a halting site with caravans, and five additional images showed caravans in the background behind individuals from the Traveller community. Further symbols identifying the distinctive elements of Traveller lifestyle included three images of horses and carriages and two images of traditional wedding attire. Considering research exploring children's awareness of Travellers is very limited, the task sought to examine whether social categorisations would also incorporate less perceptible cultural symbols such as language, sport, religious practices, political figures and events, wide family and community networks, and traditional trades of tinsmithery and horse‐trading, identified in research with adults (Bloomer et al., 2013; Boyle et al., 2020; King & O'Riordan, 2019; McElwee et al., 2003).

The images were available for non‐commercial use within the public domain and were not altered by the authors for the purposes of this study. For each pair, the Traveller and Irish image were matched for effect (e.g., all smiling), colour, age and number of people depicted. All image pairings were further refined through collaboration with our Local Advisory Committee (LAC) including the Southside Travellers Action Group (STAG) and the Irish Traveller Movement (ITM). Regular meetings were held with our LAC throughout the task design and development process. For example, the inclusion of an image pertaining to the traditional trade of tinsmithery was based on a suggestion from our STAG committee member, who subsequently provided an image of a Traveller tinsmith. The ITM representative reviewed the newsletter prior to distribution and provided insight on ways to make the findings more understandable.

#### Outgroup categorisation

Children were presented with a random subset of five of the 20 image pairs and asked which represents the Traveller community. Each child was presented with a pair of two images (1 Traveller/1 settled) for each of the five trials. The order of image pairs and side the traveller image appeared were randomised. Children's responses were coded 1 if they correctly selected the Traveller symbol (e.g., traveller politician identified as “Traveller”) and 0 if the image was incorrectly categorised (e.g., Irish language word identified as “Traveller”). A total score was calculated for each participant with a range from 0 to 5; higher scores indicated greater awareness of the symbols associated with the Traveller community.

## RESULTS

### Preliminary findings

To examine social categorisation against chance, a series of one‐sample *t*‐tests with a comparison value of 0.5 were conducted using a Bonferroni correction (.05/20 = *p* < .0025, see Table 1). As each child only saw five of the 20 possible image pairs, a traditional cut‐off value of *p* < .05 is also noted for each symbol.

**TABLE 1:** Image categorisation against chance

Category	Traveller image	Settled image	t	df	p‐value	Cohen's d
Housing	Extended family (outside caravan)	Extended family (outdoors)	6.565	32	<0.001^**^	1.14
Housing	Girl with doll (outside caravan)	Girl with doll (inside house)	6.63	37	<0.001^**^	1.08
Housing	Girls running (outside caravan)	Girls running (outdoors)	4.761	36	<0.001^**^	.78
Housing	Children with toy cars (outside caravan)	Children with toy cars (indoors)	3.769	34	<0.001^**^	.51
Culture/Housing	Bride and father (outside caravan)	Bride and father	3.439	38	<0.001^**^	.55
Culture	Traditional trade: horse and carriage	Man riding a horse	4.568	35	<0.0001^*^	.76
Housing	Caravans (halting site)	Mobile homes (camping site)	3.104	36	0.004^*^	.51
Culture	Traveller street art depicting horses	Street art in Dublin	3.028	30	0.005^*^	.54
Culture	Music: Traveller women playing traditional instruments	Women playing traditional instruments	2.661	36	0.012^*^	.46
Culture	Sport: Man boxing	Man doing martial arts	−2.092	35	0.044^*^	−.35
Culture	Religion: Boy on bike with a statue of the Virgin Mary	Boy on bike (neutral background)	−1.964	39	0.057	−.31
Politics	Traveller politician	Irish politician	1.768	33	0.086	.30
Culture	Traveller children at fair	Irish children at fair	−1.505	36	0.141	−.25
Culture	Road sign: Horse and carriages prohibited	Cattle crossing	−1.348	35	0.186	−.23
Culture	Traditional trade: Tinsmith	Blacksmith	−1.275	39	0.210	−.20
Language	Cant for “woman”	Irish for “woman”	0.842	34	0.406	.14
Politics	Traveller ethnicity pin	Celtic harp coin	0.777	40	0.442	.12
Politics	Protest for Traveller rights	Protest for education	−0.476	38	0.637	−.08
Culture	Photo of Traveller wedding party	Photo of wedding party	−0.158	38	0.875	−.03
Language	Cant for “man”	Irish for “man”	0.00	37	1.000	<.00

* *p* < .05.

** Bonferroni‐corrected *p* < .0025.

Overall, children categorised six symbols above chance at the Bonferroni significance level. Four images belonged to the housing category and contained caravans in the background, whereas the foreground depicted another person/object of interest (i.e., Traveller boys playing in toy cars, Traveller girl holding a doll, Traveller girls playing outside, family gathered outside their caravan). A fifth image was from the culture category (i.e., Traveller wedding celebration also showed a caravan in the background). The only image without caravans which sorted above chance portrayed a traveller man riding in a traditional horse and carriage. Using *p* < .05, four additional images were categorised above chance: one image from the housing category depicting caravans in a halting site without the presence of another person/object, and three images belonging to the culture category (i.e., Traveller street art showing horses, a pair of Traveller women playing fiddles, and a famous boxer from the Traveller community). Children were unable to sort the remaining 10 symbols related to politics, language and culture categories (i.e., religion, traditional trade, social events).

Furthermore, a Pearson's correlation found no significant association between age and total ethnic awareness (number of symbols categorised correctly), *r* (146) = .081, *p* = .325. This suggests that across middle childhood, awareness of symbols associated with the Traveller community remains stable (see Appendix A).

## DISCUSSION

Expanding our knowledge of social identity development theory (SIDT) and ethnic awareness (Nesdale, 2004; Quintana, 1998), we used an empirical task to quantify children's awareness of the Traveller community. This study advances understanding by exploring majority‐group children's awareness of a non‐perceptually distinctive ethnic group. SIDT proposes that ethnic awareness emerges at around age three and continues to evolve as children grow increasingly aware of physical and non‐physical distinctions between social groups (Nesdale, 2004). Inversely, the current study found no significant relationship between age and children's overall ethnic awareness (total symbols categorised as hypothesised). Across ages 6–12, symbols from the housing and culture categories were categorised above chance (e.g., caravans, a Traveller wedding, a horse and carriage). Children did not demonstrate ethnic awareness of many of the symbols associated with the politics, language, or other aspects of culture. This is consistent with previous qualitative findings (Dupont, 2017) wherein majority‐group children demonstrated limited awareness of the Traveller community beyond a basic knowledge of their nomadic lifestyle and stereotypical Traveller weddings. Together with the current study, these findings underscore the need to include Traveller culture within the primary school curriculum to enhance children's awareness of the rich and diverse elements of Traveller culture beyond a basic awareness of superficial differences.

### Limitations and future research

This study investigated children's awareness of a highly marginalised, yet traditionally understudied group. Complementing previous qualitative research, a child‐friendly task was developed in collaboration with Traveller rights organisations and tested across the primary school years. Yet, limitations could be tackled in future research. First, while the symbols selected were grounded in research and endorsed by Traveller organisations, this task was not pre‐tested with adults. Future work could explore whether the hypothesised symbols are readily categorised by adolescents and adults (e.g., McGuire et al., 2019); awareness of more subtle features (e.g., politics or trade) might only develop later. Correspondingly, as limited ethnic awareness remained stable across middle childhood (aged 6–12), future research could also pilot this task with preschool‐aged children to pinpoint when awareness of perceptually obvious markers of Traveller identity emerges.

The forced selection design of the task (i.e., Traveller/settled images presented in a comparative manner) does not reflect real‐life situations where children interact with multiple social groups simultaneously and encounter group symbols across dynamic situations which rely on different sensory pathways. Future research might explore how effectively children categorise symbols presented through different media types (e.g., static images, sound recordings, music, videos). Furthermore, the Common Ingroup Identity Model (CIIM) proposes that inducing members of different groups to view themselves as belonging to a single more inclusive group (e.g., a nation) can strengthen overall identification with the common ingroup, which in turn promotes more positive intergroup relations (Gaertner & Dovidio, 2012). As majority‐group children and Travellers are both native to Ireland and share features of the dominant culture, children in this study may have struggled to differentiate between symbols associated with the common Irish identity. To explore common ingroup identity models, the task could be modified to incorporate distractor images (e.g., stick figures) to determine whether these neutral images influence the rate of children's identification with the Traveller image to the same degree as the settled Irish images.

The reliance on convenience sampling due to the pandemic was a further limitation. Travellers are more urbanised than the general population and tend to live separately from the settled Irish within close‐knit communities (Central Statistics Office, 2016); thus, it is probable that children in this study would have had limited contact with Traveller children. While we did not record the geographic location of individual participants, three items were administered to measure children's contact (quantity, quality and friendship) with Travellers. Considering the COVID‐19 pandemic, children were asked to think about both contact quality and quantity in hypothetical terms. There were no significant associations between the total number of Traveller images categorised correctly and the three contact items. This corresponds to reports that a relatively small number of settled people have personal contact with Travellers (Kavanagh & Dupont, 2021; Appendix B). Future research should explore the influence of intergroup contact on children's ethnic awareness by adopting a stratified sampling approach to include children living in cities (e.g., Cork, Galway) and towns (e.g., Tuam, Longford, Dundalk) with higher populations of Travellers (Central Statistics Office, 2016). Finally, using the “screensharing” feature of Zoom to record children's awareness of a traditionally marginalised group may have resulted in social desirability bias. Future work should investigate whether a face‐to‐face test, wherein children complete the task privately without the experimenter's assistance (e.g., using an iPad), would influence self‐reported awareness of ethnic outgroup symbols.

### Implications for policy and practice

Schools are in a key position to educate children about cultural identity and belonging (Devine et al., 2008). The current study revealed that ethnic awareness of Traveller symbols did not increase across the primary school years; that is, children's categorisation relied on perceptually obvious markers of Traveller ethnicity. The lack of age‐related differences in knowledge of Traveller identity underscores the need for system‐wide curriculum reform and suggests that school‐based interventions could commence as early as 6 years of age.

The recent “Traveller Culture and History in Education Bill 2018” aims to address institutional discrimination by way of curricular non‐recognition and misrecognition of Traveller culture (Kavanagh & Dupont, 2021). The inclusion of Traveller culture and history across the primary and post‐primary curricula (NCCA, 2019) should incorporate nuanced perspectives exploring the historical language of “Cant,” traditional trade, Traveller influences on Irish music and folklore, alongside the role of Travellers in world wars and Ireland's struggle for independence. The proposed curricula should address the fluidity and heterogeneity of culture, recognise the role of discrimination in Travellers' life experiences, and be developed and implemented with the active support of Travellers (Kavanagh & Dupont, 2021). Correspondingly, the “Yellow Flag Programme,” developed by the Irish Traveller Movement (ITM) (NCCA, 2019), offers demonstrated efficacy in helping children appreciate the importance of diversity and equality beyond the classroom setting and in everyday life (Titley, 2009). As a whole‐school approach, the Yellow Flag Programme may be the first step towards meaningful inclusion and tackling anti‐Traveller racism (Kavanagh & Dupont, 2021).

The quantitative task adapted for this study could be used to evaluate pre/post changes in children's ethnic awareness of Travellers following curriculum reform or the Yellow Flag Programme. SIDT proposes that children's development of ethnic awareness does not automatically instigate hostility towards the outgroup; instead, prejudice development depends on external factors (e.g., intergroup contact, parental and peer influences) (Nesdale, 2004). Supporting current school‐based efforts to promote knowledge of Traveller ethnicity, this task could be used to assess post‐intervention effects on children's awareness of the more nuanced features of Traveller ethnicity, beyond a reliance on stereotypical features.

Exploring ethnic awareness could help uncover the foundation of intergroup attitudes and behaviours. Although the Traveller community are an understudied population, this task could also be adapted for use in other countries with native minority groups (e.g., Canada, USA, Australia, New Zealand). Beyond physical differences, this task could uncover the influence of the historical, social and cultural symbols on majority‐group children's social categorisations of indigenous groups. Moreover, our task is applicable to the study of other vulnerable minorities including refugees, who are likely to remain a salient group in the near future.

## CONCLUSION

Limited ethnic awareness across middle childhood supports addressing Traveller history and culture in the primary school curriculum. Despite limitations, the present findings add to the literature exploring majority‐group children's awareness of the Traveller community in Ireland.

## Endnotes

^1^
One‐way between‐groups analyses were conducted to explore children's scores on the study variables across the six experimenters. There was only one significant experimenter effect on outgroup image categorisation. A post hoc analysis using Scheffe revealed that the source of this difference was only between experimenters 1 and 6. Results were then re‐run removing experimenter 6's data and the overall pattern remained the same.

## APPENDIX A

### A.1

Age differences between those who sorted each image pair correctly versus incorrectly.

**Table:**

Category	Traveller image	Settled image	t	df	p‐value
Housing	Extended family (outside caravan)	Extended family (outdoors)	−0.1.6	31	.120
Housing	Girl with doll (outside caravan)	Girl with doll (inside house)	−1.130	36	.266
Housing	Girls running (outside caravan)	Girls running (outdoors)	−0.644	35	.524
Housing	Children with toy cars (outside caravan)	Children with toy cars (indoors)	−1.400	33	.171
Culture/Housing	Bride and father (outside caravan)	Bride and father	−0.263	37	.794
Culture	Traditional trade: Man with horse and carriage	Man riding a horse	−2.199	34	.035^*^
Housing	Caravans (halting site)	Mobile homes (camping site)	−1.946	35	.060
Culture	Traveller street art depicting horses	Street art in Dublin	0.773	29	.446
Culture	Music: Traveller women playing traditional instruments	Women playing traditional instruments	0.105	35	.917
Culture	Sport: Man boxing	Man doing martial arts	−0.976	34	.336
Culture	Religion: Boy on bike with a statue of the Virgin Mary	Boy on bike (neutral background)	−1.711	38	.095
Politics	Traveller politician	Irish politician	−0.151	32	.881
Culture	Traveller children at fair	Irish children at fair	−0.699	35	.489
Culture	Road sign: Horse and carriages prohibited	Cattle crossing	0.806	34	.426
Culture	Traditional trade: Tinsmith	Blacksmith	0.830	38	.412
Language	Cant for “woman”	Irish for “woman”	0.086	33	.932
Politics	Traveller ethnicity pin	Celtic harp coin	−0.214	39	.832
Politics	Protest for Traveller rights	Protest for education	1.014	37	.317
Culture	Photo of Traveller wedding party	Photo of wedding party	0.550	37	.586
Language	Cant for “man”	Irish for “man”	−0.970	36	.339

## APPENDIX B

### B.1

Traveller definition, contact measures and analyses.

***Contact***

Once children had completed both the outgroup categorisation and ingroup symbol preference tasks, the experimenter read them the child‐friendly definition of the Traveller community:

***Who are Travellers?***
Travellers are a community of Irish people who have been living in Ireland for hundreds of years. Over these years, travellers have shared their own history, language, culture, and traditions. In the past, Travellers would move all over Ireland in caravans. Today, some Travellers still travel all around the country, while a lot of other Travellers stay in the one place and live in houses or caravans.

Next, three items were used to measure children's contact with Travellers. Higher scores on each of these individual items were indicative of more positive tendencies for contact.

**Hypothetical Contact Quantity.** Children were shown a scale of five balloons of various sizes and asked: “Before the outbreak of COVID‐19, which balloon shows how much time you would like to spend with Traveller children?”. Responses ranged from 0 (none) to 4 (all of my time).

**Friendship Aspect of Contact.** Children were asked: “How many close friends do you have that are Travellers?”. Responses ranged from 0 (none) to 3 (all) in a scale of four ascending balloons.

**Hypothetical Contact Quality**: A scale of four hands indicating a range from thumbs up to thumbs down was used to measure hypothetical contact quality. Children were asked: “How good or bad do you think your experiences would be with Traveller children?”, with responses ranging from 0 (always bad) to 3 (always good).

Means, standard deviations and bivariate correlations of contact measures and number of Traveller images categorised correctly (*N* = 148).

**Table:**

Variables	M	SD	Range	1.	2.	3.	4.
1. Images categorised correctly	3.014	1.154	0–5	—	—	—	—
2. Hypothetical Contact Quantity	1.94	1.018	0–4	−.150	‐	—	—
3. Contact (friendship)	0.21	.457	0–3	−.065	.175^*^	—	—
4. Hypothetical Contact Quality	2.07	0.748	0–3	−.064	.435^**^	.014	—

* Correlation is significant at 0.05 level (two‐tailed).

** Correlation is significant at 0.01 level (two‐tailed).

## APPENDIX C

### C.1

Example of open‐source image pairs.Which picture represents the traveller community? (Politics: travelle/settled politicians) (Traveller Politician https://commons.wikimedia.org/wiki/File: Eileen_Flynn_2020.jpg. Irish Politician https://commons.wikimedia.org/wiki/File: Holly_Cairns.jpg)

Which picture represents the traveller community? (Culture: traditional trade). (Traveller Craft‐ Image provided by Local Advisory Committee. Settled Craft https://www.geograph.ie/photo/4808094
